# Bleeding at the Nose

**Published:** 1886-05

**Authors:** 


**Bleeding at the Nose.**—A correspondent of the Scientific Americans says: "The best remedy for bleeding at the nose, as given by Gleason in one of his lectures, is a vigorous motion of the jaws, as if in the act of mastication. In the case of a child a wad of paper should be placed in its mouth, and the child instructed to chew it hard. It is the motion of the jaws that stops the flow of blood. This remedy is so very simple that many will feel inclined to laugh at it; but it has never been known to fail—not even in very severe cases.

